# Orthopaedic and trauma research in Tanzania: A scoping review

**DOI:** 10.1371/journal.pone.0304218

**Published:** 2024-06-05

**Authors:** Benjamin Blackman, Sarah Barnett, Ajay Premkumar, Neil P. Sheth

**Affiliations:** 1 School of Medicine, University of Limerick, Limerick, Ireland; 2 Perelman School of Medicine, University of Pennsylvania, Philadelphia, Pennsylvania, United States of America; 3 Department of Orthopaedic Surgery, Emory University School of Medicine, Atlanta, Georgia, United States of America; 4 Department of Orthopaedic Surgery, Philadelphia, Pennsylvania, United States of America; University Hospital Zurich, SWITZERLAND

## Abstract

Tanzania is disproportionately burdened by musculoskeletal injuries as it faces unique challenges when dealing with trauma care. This scoping review aims to summarize and assess the current state of orthopaedic and trauma research in Tanzania. By identifying key themes, trends, and gaps in the literature, this review seeks to guide future research initiatives catered specifically to the needs of Tanzania’s healthcare system. Utilizing the PRISMA-ScR protocol, OVID Medline, PubMed, and CINAHL databases were searched from inception to June 17, 2023, using keywords such as “Orthopaedics” “Trauma” and “Tanzania”. One hundred and ninety-two eligible studies were included and the Arksey and O’Malley framework for scoping studies was followed. There was a notable growth of relevant publications from 2015 onward, with peaks in growth in the years 2019, 2021, and 2020. The studies employed diverse research methodologies, with cross-sectional (n = 41, 21%) and prospective studies (n = 39, 20%) being the most prevalent, and randomized-controlled trials being the least prevalent methodology, making up eight studies (4.2%). The most common study themes were trauma (n = 101, 52.6%), lower extremity (n = 31, 16%), and spine-related issues (n = 27, 14%). Only three studies looked at work-related injuries (1.6%). Road traffic injuries (RTIs) were the most common mechanism of trauma in 77.0% of the trauma focused studies. Fifty-three percent of the studies were conducted by a majority of Tanzanian authors. This scoping review highlights various trends in orthopaedic and trauma research in Tanzania, with a particular emphasis on road traffic-related injuries. Various gaps are explored, including a lack of research on work-related injuries and a paucity of experimental research. Our findings underline areas where future research is warranted. The future of orthopaedic and trauma care in Tanzania depends on the efforts and collaboration of both local and international stakeholders.

## Introduction

Injuries make up 9% of global deaths, with 90% occurring in low and middle-income countries (LMIC) [1]. Alongside other developing countries, Tanzania faces unique challenges when dealing with orthopaedic and trauma care [2].

With a rapidly growing population and increased economic development, orthopaedic injuries continue to rise markedly, mainly due to road traffic injuries (RTI). In a one-day survey conducted in Tanzania, nearly half of all trauma cases were attributed to RTIs [3]. While the need for orthopaedic care is on the rise, there is a lack of available orthopaedic services and an insufficient number of adequately trained healthcare professionals in the field [4]. Limited access to surgical equipment and inadequate infrastructure for the volume of required care [5] renders orthopaedic and trauma management a significant strain on the country of Tanzania.

Multiple reports have highlighted the need for local healthcare research to improve health outcomes in developing countries [6, 7]. Furthermore, local research in developing countries has been shown to be of utmost importance in contributing to infrastructural growth [8–10]. In Tanzania, the significance of generating robust local research becomes particularly pronounced due to persistent obstacles in the provision of adequate trauma care [11, 12].

By employing a scoping review approach, this study aims to comprehensively survey and synthesize the existing orthopaedic and trauma literature within the context of Tanzania. This methodology facilitates the examination of a wide spectrum of literature by date of publication, research methodologies employed, and overarching themes addressed. This thorough assessment provides a comprehensive summary of the existing literature, as well as a foundational platform for future research endeavors. Moreover, this review seeks to align its findings with the unique requirements of Tanzania’s healthcare system, enhancing its capacity to contribute meaningfully to the advancement of orthopaedic and trauma care in the region.

## Methods

A scoping review was chosen due to the breadth of the research topic and the expected variation in study design, and was conducted using the Arksey and O’Malley framework [13].

### Identification of the research question

Our research question was, “What is the current state of orthopaedic and trauma literature in Tanzania, and where should future research be directed?”

### Protocol

The preferred reporting items for systematic reviews and meta-analyses extension for scoping reviews (PRISMA-ScR) protocol was used to present the study methodology and findings (S1 Checklist) [14].

### Identification of the relevant studies

The authors executed a search using the following databases: OVID Medline, PubMed, and CINAHL using controlled vocabulary (e.g. MeSH) and keywords representing the topics “Orthopaedics”, “Trauma” and “Tanzania”.

Databases were searched from inception to June 17, 2023. With the goal of capturing the entirety of the relevant existing literature, no limits were applied. Results (n = 1573) were exported to Rayyan citation management system [15]. A detailed search strategy is available in S1 File.

### Study selection

Two co-authors independently screened titles and abstracts of identified papers. After full-text screening by each reviewer, papers were categorized into retrospective cohort, prospective cohort, cross-sectional studies, case studies and reviews, randomized controlled trials, and other research (for e.g., cost analysis, discussion, mixed-method). Full-text extraction was then carried out using a data extraction sheet developed for the purpose of this study. Each author verified that the papers met the inclusion criteria and focused on the topic of interest. Discrepancies in reviewers’ decisions were resolved through discussion.

### Inclusion and exclusion criteria

Studies met the inclusion criteria if they met the following criteria: (1) Study focused on orthopaedic conditions or injuries suffered from trauma, and (2) Study reported findings from a Tanzanian population.

### Data charting

The same two co-authors independently reviewed each paper, discussed charted data, and updated a password protected Google Sheets [16] datasheet accordingly. Information extracted from the selected studies was organized and categorized as follows: authors and publication date, study type, study topic, and study context. Studies pertaining to trauma were further categorized by mechanism of injury from RTIs, falls, and violence, where the percentage of patients from each category was documented. Additionally, the country of origin pertaining to authorship was charted.

### Collating and summarizing findings

A thematic data synthesis was performed to identify the state of orthopaedic literature in Tanzania. The synthesis includes useful information on past research focus, existing gaps, and suggested future initiatives.

Due to the heterogeneity of study designs and outcomes, a narrative summary of results is presented.

## Results

### Search results

**Fig 1:**
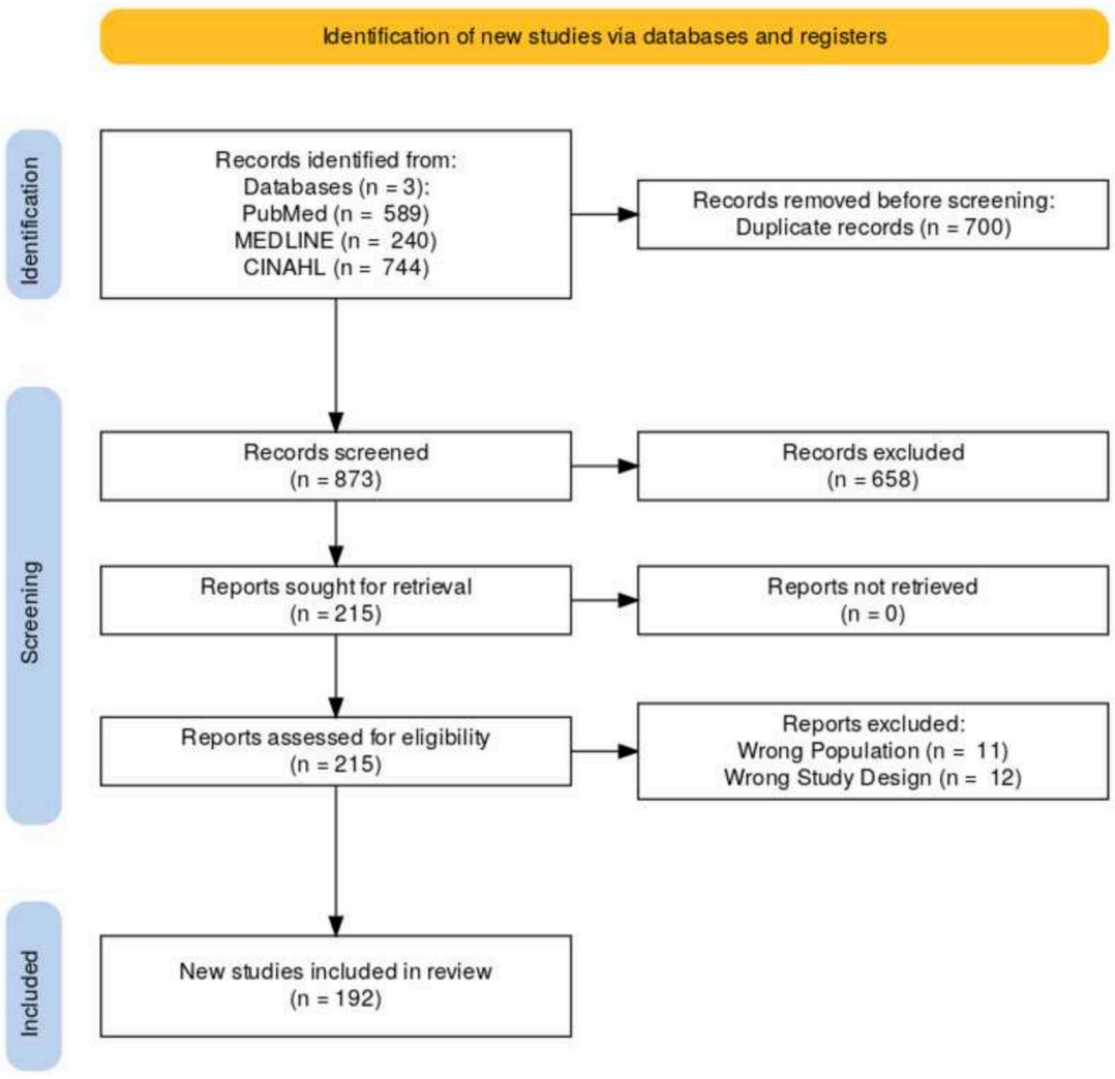
PRISMA flow chart.

A preliminary search of scientific databases yielded a total of 1,573 studies. After removing the duplicates (n = 700), titles and abstracts of 873 studies were screened. This process excluded an additional 658 studies, leaving a sample of 215 studies. Screening of full texts yielded a total sample of 192 studies eligible for this review. A complete PRISMA study flow diagram is shown in Fig 1.

### Distribution of studies by year

**Fig 2:**
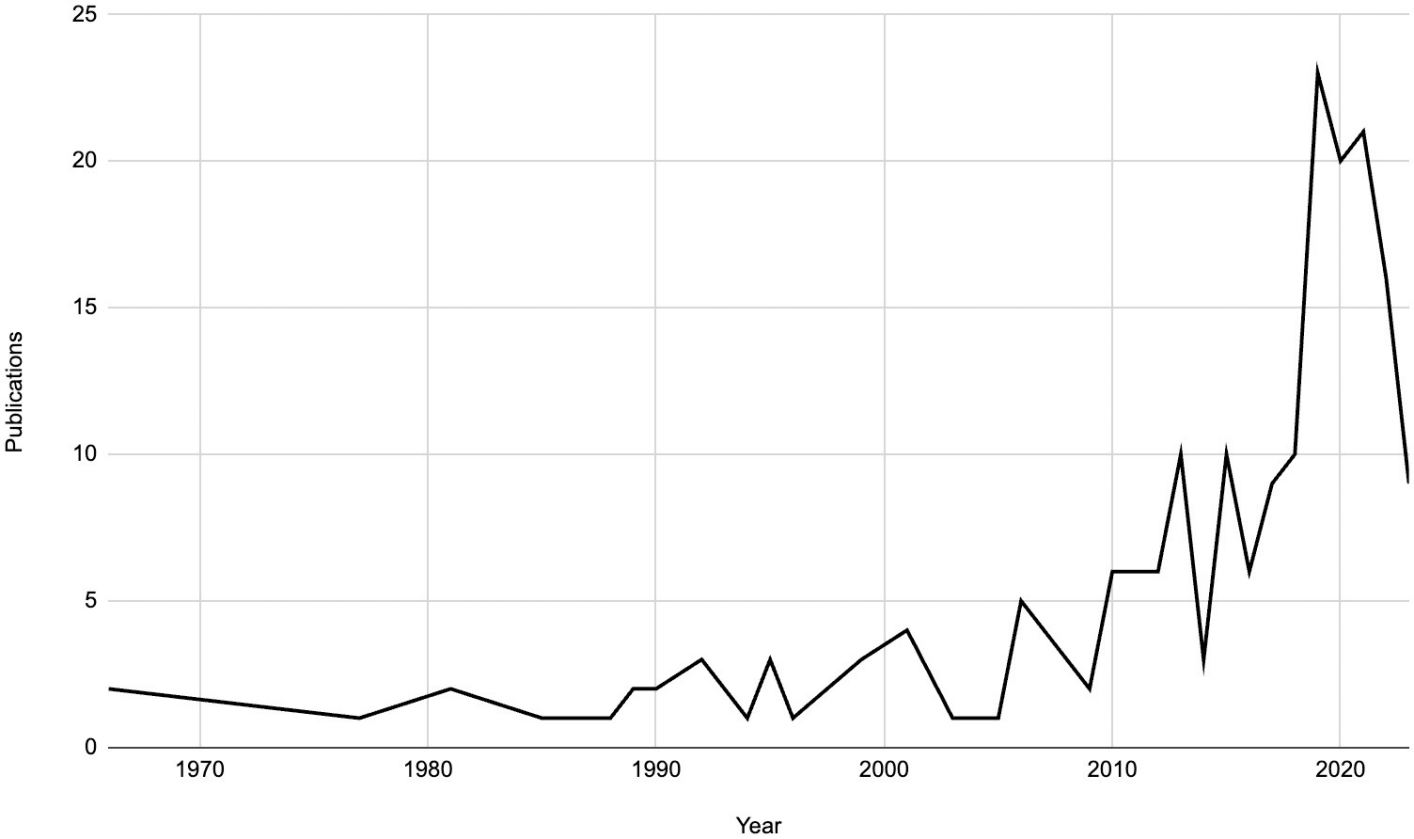
Publications per year.

There were no limits placed on the dates of studies included, as this scoping review aimed to capture all the relevant literature on orthopaedics and trauma in Tanzania. As a result, several decades of research were examined, dating back to 1966. Since then, the body of research grew sporadically, with the early 2000s showing a relatively steady increase. From 2015 onwards, there was a significant increase in published studies in this field. The years 2019, 2021, and 2020 marked the peaks in the literature, during which 23, 21, and 20 studies were published, respectively. In 2022, there were 16 studies, and in 2023, there have been 9 studies to date (Fig 2).

### Research methodology

The scoping review examination of orthopaedic research and trauma care in Tanzania yielded diverse research methodologies that represented the multifaceted field of inquiry. The studies were primarily categorized as follows: retrospective cohort studies (14), cross-sectional studies (41), prospective studies (39), case studies (24), retrospective reviews (34), case series (9) and randomized controlled trials (8). Twenty-six studies fell in the category of other research: cost analysis (5), survey (4), qualitative (4), discussion (3), descriptive (3), mixed-method (3), modelling (1). A breakdown of study methodology is shown in Table 1. This diversity in study methodologies presents opportunities and challenges when reviewing the data to guide future research in Tanzania.

**Table 1:** Study types.

Study Type	Count (%)
Cross-Sectional	41 (21.4%)
Prospective	39 (20.3%)
Retrospective Reviews	34 (17.7%)
Case Studies	24 (12.5%)
Retrospective Cohort	14 (7.3%)
Case Series	9 (4.7%)
RCTs	8 (4.2%)
Other	23 (12.0%)

### Thematic analysis

Various themes were identified which highlighted the existing breadth of research in the field and identified areas where research is lacking. There were 101 trauma-specific studies, which included a focus on RTIs, falls, violence, and other trauma. Fifty-six studies focused on fractures. Overall, 31 studies looked specifically at the lower extremity and seven focused on the upper extremity. Twenty-seven studies addressed spine-related issues, and 27 studies examined a pediatric population. Fifteen studies discussed infections, eight discussed genetic conditions, and four discussed cancer. Finally, seven studies addressed health economics, and seven addressed public health. These results are depicted in Table 2.

**Table 2:** Study themes.

Themes	Count (%)
Trauma	101 (52.6%)
Fractures	56 (29.1%)
Lower Extremity	31 (16.1%)
Pediatrics	27 (14%)
Spine	27 (14%)
Infection	15 (7.8%)
Genetic	8 (4.2%)
Upper Extremity	7 (3.6%)
Health Econ	7 (3.6%)
Public Health	7 (3.6%)
Cancer	4 (2.1%)
Work-Related Injuries	3 (1.6%)

### Trauma specific studies

Of the 101 trauma related studies, 61 (60.3%) further explored injury mechanisms in cohorts involving multiple admission causes (RTI, falls, violence, etc.). Of these studies, the most common cause of trauma were RTIs (77.0%), followed by falls (18.0%) and violence (4.92%). Almost 97% of trauma-focused studies included patients injured in RTI, 74.2% included patients injured from falls, and 50% included patients injured because of violence (Table 3).

**Table 3:** Trauma-focused studies’ most common cause of injury.

Mechanism of Trauma	Most Common Cause (%)
Road Traffic Injuries	47 (77%)
Falls	11 (18%)
Violence	3 (4.92%)

### Randomized controlled trials analysis

Of the eight studies classified in the category of RCT, there were four unique trials, one of which is in progress. There were five studies that reported results from RCTs [17, 18, 22–24], two RCT protocols [19, 20], and one pilot study [21]. Four of the eight studies focused on infection prevention [17–20]. In 1989, Museru, Kumar, and Ickler compared isotonic saline, distilled water, and boiled water in irrigation of open fractures, and found no difference in outcomes [17]. In 2015, Marwa et al. examined the use of cefepime versus ceftriaxone prophylaxis in elective orthopaedic procedures and found no significant difference [18]. Two protocols were published with reference to the Go-Tibia trial, which aims to be completed by 2028 [19, 20]. This trial is a masked randomized control trial assessing the rate of infection after gentamicin or saline administration in patients with open tibia fractures. The other four included RCTs related to a prospective trial comparing intramedullary nailing versus external fixation in the treatment of open tibial fractures [21–24]. The pilot study was published in 2018 [21], which was followed by a cost effectiveness analysis in 2019 [22], which revealed that intramedullary nailing was more cost effective and had better union rates at three months follow-up. The following publication at a one-year follow-up showed no difference in primary events, however found that intramedullary nailing yielded better coronal alignment [23]. A three to five year follow up of this cohort revealed that 25% of patients suffered chronic fracture-related infection and non-union, regardless of reintervention [24].

### Work related injuries

There were three studies focusing specifically on work-related injuries. In 2010, Kishashu et al. surveyed 1385 patients with injuries from 2007 to 2008 and found that 638 (46%) were work related [25]. In 2013, Boniface et al. examined 248 miners suffering WRI from 2009 to 2012 and found that 98.7% of workers did not use protective gear and worked over 12 hours daily. Falling rocks were the leading cause of injury, and in total 41.3% of these patients died [26]. In 2021, Shewiyo et al. performed a retrospective review of 4578 claims to the Workers Compensatory Fund in Tanzania from 2016 to 2019. They concluded that motor accidents, machine faults, and falls were the most common causes of WRI. They also reported the odds of a work-related fatalities increased greater than 6-fold in injuries occurring during conveyance [27].

### Country of origin

Of the 192 studies included, 91% had Tanzanian authors and 53% had a majority of Tanzanian authors. Of the studies with a majority of out-of-county authors, 55% of these studies had a majority of authors from the United States. Further, the USA was the most represented country after Tanzania, with 40% of all included studies including an American author. Africa was the most represented continent (54%), followed by North America (26%) and Europe (10.4%).

## Discussion

This scoping review outlines the evolution of orthopaedic trauma care in Tanzania, as reflected in the distribution of studies spanning several decades. The growth in orthopaedic and trauma research in Tanzania since 1966 is noteworthy as it represents shifts in scientific, societal, and health policy interests. The early years of research were marked by subtle and inconsistent growth, indicating the lower priority of trauma care in Tanzania. This is likely because infectious diseases such as HIV/AIDS, malaria, and tuberculosis had posed a more imminent threat to public health [28]. In particular, the last decade marked a significant period of growth for orthopaedic and trauma research. This may be attributed to increased awareness and international collaboration among world experts [29, 30].

### Road traffic injuries

The predominant focus on RTIs among trauma cases reveals the substantial burden created by these injuries. The increased incidence of RTIs is likely a result of the exponential proliferation of roads, which led to underdeveloped and unsafe driving conditions [31, 32]. Roughly 10% of the roads in Tanzania are paved [33]. The increase in RTIs is multi-factorial and has increased due to more motorized vehicle use (e.g., cheaper foreign motorcycles from China and India) in conjunction with a lack of proper infrastructure (e.g., lack of sidewalks, lights on roads, road quality) and inadequate safety measures (e.g., seatbelt and helmet laws, traffic lights, stop signs) [34–36]. In 2011, the World Health Organization (WHO) and the World Bank launched a Decade of Action for Road Safety with the goal of cutting RTIs and deaths in half [37]. However, minimal progress has been made in LMICs [38], underscoring the need for further preventative initiatives.

Addressing road safety remains of utmost importance for the Tanzanian population. Ahmed et al. (2013) report that raising awareness of RTIs is integral in low-income countries [39]. They recommend increasing public awareness, road safety infrastructure, and traffic rules as the most effective means for reducing traffic accidents [39]. Thus, further research on the outcomes of these initiatives, and the formulation of health policy specific to the Tanzanian population, will be vital in minimizing the burden of RTIs.

In addition to raising awareness, future research should focus on rehabilitation and post-operative management of trauma patients, as this theme was seldom examined in the literature. By optimizing rehabilitation, patients will be better able to return to their livelihood, support their families, and contribute to the country’s economic growth, benefiting all shareholders [40].

### Economic impact of injury

The financial implications of injuries remain a prevalent concern in Tanzania. On average, 52% of a citizen’s total health expenditure is out-of-pocket [41]. Over a one-month period, 75% of Tanzanians surveyed in the Kilimanjaro Christian Medical Center orthopaedic ward reported that their healthcare expenditure cost more than their monthly income, and roughly 40% of these patients reported losing their job due to their disability after their injury [42]. With nearly 26 million people in Tanzania living in extreme poverty (below 1.90 U.S. dollars a day) [43], the cost of health care augments the economic strain on trauma victims.

In addition to improving infrastructure and raising awareness, addressing work-related injuries (WRI) will help alleviate the overall trauma burden. When appropriate measures are put into place, WRIs are often preventable [44]. Such measures include safe working environments, appropriate protective equipment, appropriate training, and rehabilitation initiatives [45]. Poor workplace conditions place an economic and health burden on society [45, 46]. The International Labour Organization (ILO) estimates that approximately 4% of the world’s gross domestic product is lost due to WRIs, and low-income countries are particularly affected [47, 48]. In 2015, it was estimated that, in the Southern Africa region alone, 18,000 workers died from work-related accidents [49]. In Tanzania, WRI remains an unaddressed epidemic. This scoping review found a scarcity of research specifically related to WRI, with only three studies focusing on this topic. Although many of the trauma studies included patients who were injured at work, there is little research on interventions and initiatives focused on reducing injuries in the workplace.

### Sustainability

Under conservative assumptions, over 90% of the Northern Tanzania population cannot access orthopaedic surgical services [50]. Tanzania has an estimated 118 orthopaedic surgeons for a population of over 60 million [51], which equates to one orthopaedic surgeon for every 508,000 citizens. In stark contrast, this ratio in America is one orthopaedic surgeon for every 10.8 thousand citizens [52].

In recent years, there has been increased enthusiasm for medical missions to developing countries as initiatives such as Doctors without Borders continue to expand [53–55]. As international orthopaedic surgeons volunteer in Africa on medical missions, addressing the long-term sequelae of their work is vital. These mission trips often involve completing many surgeries quickly, introducing new equipment and thus, potentially new complications, which further increase the burden on the local healthcare system [30, 56]. For a more sustainable future of care, global collaboration is critical to develop adequate infrastructure for continuous orthopaedic management in Tanzania [30, 57, 58].

### Future research

Given that developing countries constitute the majority of the global population [59], conducting research tailored to these populations is imperative. As delineated by the 10/90 phenomenon, only 10% of worldwide resources are devoted to the population which suffers 90% of the burden [60]. This scoping review found eight studies pertaining to randomized controlled trials, with four being unique trials. This scarcity highlights the need for further research into the population-specific determinants of health in Tanzania. In their systematic review of orthopaedic global outreach efforts, Nolte et al. concluded that orthopaedic outreach initiatives in LMICs are cost-effective and direct funding is needed to ameliorate global orthopaedic health [61]. Of the ten studies on surgical mission trips to Africa included in their review [61], none were in Tanzania, further highlighting the need for research in this specific population.

It is well-accepted that randomized clinical trials are the gold standard for assessing causal relationships in research [62]. However, this research methodology is often expensive and time-consuming [63]. In developing countries, various barriers exist to conducting high-quality research [64], such as lack of suitable research infrastructure, deficiency in policymakers understanding of the importance of research, and absence of research materials [65–67]. Historically, research in Tanzania has not been focused on the context-specific structural determinants of health and inequities, which warrants wider implementation of local investigator-initiated trials [64, 67]. Further initiatives should focus on interdisciplinary collaboration in creating appropriate research infrastructure to better suit randomized controlled trials. As more experimental research is done in Tanzania, the management of orthopaedic conditions will be better understood to ultimately improve patient outcomes.

Most of the studies included were conducted by Tanzanian authors. Nevertheless, addressing orthopaedic and trauma care in other LMICs remains a global health priority. In LMICs, RTIs remains a significant health burden, due to similar factors discussed in this review [1]. Longitudinal partnerships and research that reflect the interests and goals of local populations in other LMICs are needed to further explore this topic, and ultimately improve global health outcomes [6–10].

### Strengths and limitations

The major strength of this scoping review is its comprehensive overview of the literature. By including studies dating back to 1966, this review encapsulates the wide range of methodologies, focuses, and themes in orthopaedic and trauma research in Tanzania. This breadth of included studies allowed for a thorough review of trends and gaps in the literature, highlighting areas where future research is warranted. The main limitations are the lack of ability to perform a meta-analysis and lack of study quality (risk of bias) assessment.

## Conclusion

The escalating orthopaedic and trauma crisis in Tanzania demands immediate attention and international intervention to mitigate the country’s massive health and economic burden. This scoping review offers a comprehensive analysis of orthopaedic and trauma research conducted in Tanzania from 1966 to 2023. Our findings highlight the prevalence of RTIs as a significant cause of injuries, emphasizing the pressing need for more effective interventions and health policies to reduce this burden. The notable scarcity of research on WRIs and randomized controlled trials indicates a significant gap in the existing literature, highlighting the need for high-quality research in these areas in Tanzania. A commitment to implementing sustainable orthopaedic and trauma care should be a local and global priority. This scoping review aims to catalyze further research endeavors and outreach initiatives in Tanzania’s orthopaedic and trauma sectors.

## Supporting information

S1 Checklist PRISMA-ScR checklist. (PDF)

S1 File Search strategy. (PDF)

## References

[1] World Health Organization. Injuries and violence: the facts 2014 [Internet]. World Health Organization; 2014 [cited 2023 Aug 24]. 19 p. https://apps.who.int/iris/handle/10665/149798

[2] Hardaker WM, Jusabani M, Massawe H, Pallangyo A, Temu R, Masenga G, et al. The burden of orthopaedic disease presenting to a tertiary referral center in Moshi, Tanzania: a cross-sectional study. Pan Afr Med J. 2022;42:96. doi: 10.11604/pamj.2022.42.96.30004 36034039 PMC9379434

[3] Sawe HR, Mfinanga JA, Mbaya KR, Koka PM, Kilindimo SS, Runyon MS, et al. Trauma burden in Tanzania: a one-day survey of all district and regional public hospitals. BMC Emergency Medicine [Internet]. 2017 Oct 13 [cited 2023 Aug 24];17(1):30. Available from: doi: 10.1186/s12873-017-0141-6 29029604 PMC5640911

[4] Premkumar A, Massawe H H., Mshabaha D J., Forans J R., Ying X, Sheth N P. The burden of orthopaedic disease presenting to a referral hospital in northern Tanzania. Glob Surg [Internet]. 2016 [cited 2023 Aug 24];2(1). Available from: http://oatext.com/The-burden-of-orthopaedic-disease-presenting-to-a-referral-hospital-in-northern-Tanzania.php

[5] Sheth NP, Hardaker WM, Zakielarz KS, Rudolph M, Massawe H, Levin LS, et al. Developing sustainable orthopaedic care in northern tanzania: an international collaboration. Journal of Orthopaedic Trauma [Internet]. 2018 Oct [cited 2023 Aug 24];32(7):S25–8. Available from: https://journals.lww.com/00005131-201810007-00008 30247396 10.1097/BOT.0000000000001296

[6] Franzen SRP, Chandler C, Lang T. Health research capacity development in low and middle income countries: reality or rhetoric? A systematic meta-narrative review of the qualitative literature. BMJ Open [Internet]. 2017 Jan 1 [cited 2023 Aug 24];7(1):e012332. Available from: https://bmjopen.bmj.com/content/7/1/e012332 28131997 10.1136/bmjopen-2016-012332 PMC5278257

[7] Acharya KP, Pathak S. Applied research in low-income countries: why and how? Front Res Metr Anal [Internet]. 2019 Nov 14 [cited 2023 Aug 24];4:3. Available from: https://www.frontiersin.org/articles/10.3389/frma.2019.00003/full 33870035 10.3389/frma.2019.00003 PMC8028400

[8] World Health Organization. Local production and technology transfer to increase access to medical devices: addressing the barriers and challenges in low- and middle-income countries [Internet]. World Health Organization; 2012 [cited 2023 Aug 24]. iv, 144 p. https://apps.who.int/iris/handle/10665/336774

[9] Minja H, Nsanzabana C, Maure C, Hoffmann A, Rumisha S, Ogundahunsi O, et al. Impact of health research capacity strengthening in low- and middle-income countries: the case of who/tdr programmes. Vlassoff C, editor. PLoS Negl Trop Dis [Internet]. 2011 Oct 11 [cited 2023 Aug 24];5(10):e1351. Available from: https://dx.plos.org/10.1371/journal.pntd.0001351 22022630 10.1371/journal.pntd.0001351 PMC3191138

[10] Sitthi-amorn C. Strengthening health research capacity in developing countries: a critical element for achieving health equity Commentary: Health research and human development in Papua New Guinea Commentary: Does strengthening research capacity improve health equity? BMJ [Internet]. 2000 Sep 30 [cited 2023 Aug 24];321(7264):813–7. Available from: https://www.bmj.com/lookup/doi/10.1136/bmj.321.7264.813 11009525 10.1136/bmj.321.7264.813 PMC1118622

[11] Fussy DS. The hurdles to fostering research in Tanzanian universities. Higher Education [Internet]. 2019 [cited 2023 Aug 24];77(2):283–99. Available from: https://www.jstor.org/stable/45116914

[12] Joiner AP, Tupetz A, Peter TA, Raymond J, Macha VG, Vissoci JRN, et al. Barriers to accessing follow up care in post-hospitalized trauma patients in Moshi, Tanzania: A mixed methods study. PLOS Global Public Health [Internet]. 2022 Jun 13 [cited 2023 Aug 24];2(6):e0000277. Available from: https://journals.plos.org/globalpublichealth/article?id=10.1371/journal.pgph.0000277 36962378 10.1371/journal.pgph.0000277 PMC10021180

[13] Arksey H, O’Malley L. Scoping studies: towards a methodological framework. International Journal of Social Research Methodology [Internet]. 2005 Feb [cited 2023 Aug 28];8(1):19–32. Available from: http://www.tandfonline.com/doi/abs/10.1080/1364557032000119616

[14] Tricco AC, Lillie E, Zarin W, O’Brien KK, Colquhoun H, Levac D, et al. Prisma extension for scoping reviews (Prisma-scr): checklist and explanation. Ann Intern Med [Internet]. 2018 Oct 2 [cited 2023 Aug 28];169(7):467–73. Available from: https://www.acpjournals.org/doi/10.7326/M18-0850 30178033 10.7326/M18-0850

[15] Rayyan—ai powered tool for systematic literature reviews [Internet]. 2021 [cited 2023 Aug 28]. https://www.rayyan.ai/

[16] Google sheets: online spreadsheet editor | google workspace [Internet]. [cited 2023 Aug 28]. https://www.facebook.com/GoogleDocs/

[17] Museru LM, Kumar A, Ickler P. Comparison of isotonic saline, distilled water and boiled water in irrigation of open fractures. International Orthopaedics [Internet]. 1989 Sep [cited 2024 Apr 14];13(3):179–80. Available from: http://link.springer.com/10.1007/BF00268044 2599691 10.1007/BF00268044

[18] Marwa JM, Ngayomela IH, Seni J, Mshana SE. Cefepime versus Ceftriaxone for perioperative systemic antibiotic prophylaxis in elective orthopedic surgery at Bugando Medical Centre Mwanza, Tanzania: a randomized clinical study. BMC Pharmacol Toxicol [Internet]. 2015 Dec [cited 2024 Apr 14];16(1):42. Available from: http://www.biomedcentral.com/2050-6511/16/42 26699529 10.1186/s40360-015-0039-4 PMC4690262

[19] von Kaeppler EP, Donnelley C, Ali SH, Roberts HJ, Ibrahim JM, Wu HH, et al. A study protocol for a pilot masked, randomized controlled trial evaluating locally-applied gentamicin versus saline in open tibia fractures (Pgo-tibia) in dar es salaam, tanzania. Pilot Feasibility Stud [Internet]. 2021 Feb 10 [cited 2024 Apr 14];7:47. Available from: https://www.ncbi.nlm.nih.gov/pmc/articles/PMC7874655/ 33568230 10.1186/s40814-021-00766-7 PMC7874655

[20] Haonga BT, O’Marr JM, Ngunyale P, Ngahyoma J, Kessey J, Sasillo I, et al. GO-Tibia: a masked, randomized control trial evaluating gentamicin versus saline in open tibia fractures. Trials [Internet]. 2023 Jun 15 [cited 2024 Apr 14];24(1):406. Available from: doi: 10.1186/s13063-023-07410-0 37322521 PMC10268448

[21] Ibrahim J, Liu M, Yusi K, Haonga B, Eliezer E, Shearer DW, et al. Conducting a randomized controlled trial in tanzania: institute for global orthopaedics and traumatology and the muhimbili orthopaedic institute. Journal of Orthopaedic Trauma [Internet]. 2018 Oct [cited 2024 Apr 14];32:S47. Available from: https://journals.lww.com/jorthotrauma/fulltext/2018/10007/conducting_a_randomized_controlled_trial_in.13.aspx 30247401 10.1097/BOT.0000000000001294

[22] Haonga BT, Areu MMM, Challa ST, Liu MB, Elieza E, Morshed S, et al. Early treatment of open diaphyseal tibia fracture with intramedullary nail versus external fixator in Tanzania: Cost effectiveness analysis using preliminary data from Muhimbili Orthopaedic Institute. SICOT J [Internet]. [cited 2024 Apr 14];5:20. Available from: https://www.ncbi.nlm.nih.gov/pmc/articles/PMC6572994/10.1051/sicotj/2019022 PMC6572994 31204649

[23] Haonga BT, Liu M, Albright P, Challa ST, Ali SH, Lazar AA, et al. Intramedullary nailing versus external fixation in the treatment of open tibial fractures in tanzania. J Bone Joint Surg Am [Internet]. 2020 May 20 [cited 2024 Apr 14];102(10):896–905. Available from: https://www.ncbi.nlm.nih.gov/pmc/articles/PMC7508278/32028315 10.2106/JBJS.19.00563 PMC7508278

[24] Cortez A, Urva M, Haonga B, Donnelley CA, von Kaeppler EP, Roberts HJ, et al. Outcomes of intramedullary nailing and external fixation of open tibial fractures: three to five-year follow-up of a randomized clinical trial. JBJS [Internet]. 2022 Nov 2 [cited 2024 Apr 14];104(21):1877. Available from: https://journals.lww.com/jbjsjournal/abstract/2022/11020/outcomes_of_intramedullary_nailing_and_external.3.aspx 35980080 10.2106/JBJS.22.00016

[25] Kishashu YM, Franzblau A, Robins T, Smith G. Patterns of severe work-related injuries in Tanzania: call for new approach to workers protection in developing countries. Injury Prevention [Internet]. 2010 Sep 1 [cited 2024 Apr 14];16(Supplement 1):A91–A91. Available from: https://injuryprevention.bmj.com/lookup/doi/10.1136/ip.2010.029215.328

[26] Boniface R, Museru L, Munthali V, Lett R. Occupational injuries and fatalities in a tanzanite mine: Need to improve workers safety in Tanzania. Pan Afr Med J [Internet]. 2013 [cited 2024 Apr 14];16. Available from: http://www.panafrican-med-journal.com/content/article/16/120/full/10.11604/pamj.2013.16.120.3420 PMC3998898 24778757

[27] Reynolds TA, Stewart B, Drewett I, Salerno S, Sawe HR, Toroyan T, et al. The impact of trauma care systems in low- and middle-income countries. Annu Rev Public Health [Internet]. 2017 Mar 20 [cited 2024 Apr 14];38(1):507–32. Available from: https://www.annualreviews.org/doi/10.1146/annurev-publhealth-032315-021412 28125389 10.1146/annurev-publhealth-032315-021412

[28] Chum HJ, OʼBrien RJ, Chonde TM, Graf P, Rieder HL. An epidemiological study of tuberculosis and HIV infection in Tanzania, 1991–1993: AIDS [Internet]. 1996 Mar [cited 2023 Aug 28];10(3):299–310. Available from: http://journals.lww.com/00002030-199603000-00009 8882670 10.1097/00002030-199603000-00009

[29] Hardaker WM, Jusabani M, Massawe H, Pallangyo A, Temu R, Masenga G, et al. The burden of orthopaedic disease in sub-saharan africa: a focus on tanzania [Internet]. In Review; 2021 Apr [cited 2023 Aug 28]. https://www.researchsquare.com/article/rs-402380/v1

[30] Sheth NP, Donegan DJ, Foran JRH, Sugarman J. Global health and orthopaedic surgery—A call for international morbidity and mortality conferences. International Journal of Surgery Case Reports [Internet]. 2015 [cited 2023 Aug 28];6:63–7. Available from: https://linkinghub.elsevier.com/retrieve/pii/S221026121400426X 25524304 10.1016/j.ijscr.2014.11.074 PMC4334206

[31] Alamgir M, Campbell MJ, Sloan S, Goosem M, Clements GR, Mahmoud MI, et al. Economic, socio-political and environmental risks of road development in the tropics. Current Biology [Internet]. 2017 Oct [cited 2023 Aug 28];27(20):R1130–40. Available from: https://linkinghub.elsevier.com/retrieve/pii/S0960982217311077 29065299 10.1016/j.cub.2017.08.067

[32] Walelign SZ, Nielsen MR, Jacobsen JB. Roads and livelihood activity choices in the greater serengeti ecosystem, tanzania. Muboko N, editor. PLoS ONE [Internet]. 2019 Mar 8 [cited 2023 Aug 28];14(3):e0213089. Available from: https://dx.plos.org/10.1371/journal.pone.0213089 10.1371/journal.pone.0213089 PMC6407761 30849100

[33] Clyde & Co LLP—Peter Kasanda. Lexology. 2014 [cited 2023 Sep 4]. An overview of the road infrastructure in Tanzania. https://www.lexology.com/library/detail.aspx?g=ba8c3064-6a64-4759-befc-2fecc5834b31

[34] Walugembe F, Levira F, Ganesh B, Lwetoijera DW. A retrospective study on the epidemiology and trends of road traffic accidents, fatalities and injuries in three Municipalities of Dar es Salaam Region, Tanzania between 2014–2018. Pan Afr Med J [Internet]. 2020 May 20 [cited 2023 Aug 28];36. Available from: http://www.panafrican-med-journal.com/content/article/36/24/full/10.11604/pamj.2020.36.24.21754 PMC7388614 32774601

[35] Chalya PL, Mabula JB, Ngayomela IH, Kanumba ES, Chandika AB, Giiti G, et al. Motorcycle injuries as an emerging public health problem in Mwanza City, north-western Tanzania. Tanzan J Health Res. 2010 Oct;12(4):214–21. 24409627

[36] Kumar A. Understanding the emerging role of motorcycles in african cities: a political economy perspective. 2011 Apr [cited 2023 Sep 4]; http://hdl.handle.net/10986/17804

[37] Second Decade of Action for road safety [Internet]. [cited 2023 Aug 28]. https://www.who.int/teams/social-determinants-of-health/safety-and-mobility/decade-of-action-for-road-safety-2021-2030

[38] Bonnet E, Lechat L, Ridde V. What interventions are required to reduce road traffic injuries in Africa? A scoping review of the literature. Shah TI, editor. PLoS ONE [Internet]. 2018 Nov 30 [cited 2023 Aug 28];13(11):e0208195. Available from: https://dx.plos.org/10.1371/journal.pone.0208195 30500856 10.1371/journal.pone.0208195 PMC6267971

[39] Ahmed SK, Mohammed MG, Abdulqadir SO, El‐Kader RGA, El‐Shall NA, Chandran D, et al. Road traffic accidental injuries and deaths: A neglected global health issue. Health Science Reports [Internet]. 2023 May [cited 2023 Aug 28];6(5):e1240. Available from: https://onlinelibrary.wiley.com/doi/10.1002/hsr2.1240 37152220 10.1002/hsr2.1240 PMC10154805

[40] Neill R, Shawar YR, Ashraf L, Das P, Champagne SN, Kautsar H, et al. Prioritizing rehabilitation in low- and middle-income country national health systems: a qualitative thematic synthesis and development of a policy framework. International Journal for Equity in Health [Internet]. 2023 May 17 [cited 2023 Aug 28];22(1):91. Available from: doi: 10.1186/s12939-023-01896-5 37198596 PMC10189207

[41] World health statistics 2013 [Internet]. [cited 2023 Aug 28]. https://www.who.int/publications-detail-redirect/9789241564588

[42] Davey S, Bulat E, Massawe H, Pallangyo A, Premkumar A, Sheth N. The economic burden of non-fatal musculoskeletal injuries in northeastern tanzania. Annals of Global Health [Internet]. 2019 Mar 4 [cited 2023 Aug 28];85(1):23. Available from: https://annalsofglobalhealth.org/articles/10.5334/aogh.1355 30873794 10.5334/aogh.1355 PMC6997525

[43] Statista [Internet]. [cited 2023 Aug 28]. Tanzania: people in extreme poverty 2016–2025. https://www.statista.com/statistics/1230404/number-of-people-living-in-extreme-poverty-in-tanzania/

[44] Nordberg E. Injuries as a public health problem in sub-Saharan Africa: epidemiology and prospects for control. East Afr Med J. 2000 Dec;77(12 Suppl):S1–43. 12862115

[45] Abdalla S, Apramian SS, Cantley LF, Cullen MR. Occupation and risk for injuries. In: Mock CN, Nugent R, Kobusingye O, Smith KR, editors. Injury Prevention and Environmental Health [Internet]. 3rd ed. Washington (DC): The International Bank for Reconstruction and Development / The World Bank; 2017 [cited 2023 Aug 28]. http://www.ncbi.nlm.nih.gov/books/NBK525209/30212110

[46] Shewiyo BS, Mwanga HH, Mrema EJ, Mamuya SH. Work-related injuries reported toworkers compensation fund in tanzania from 2016 to 2019. IJERPH [Internet]. 2021 Aug 30 [cited 2023 Aug 28];18(17):9152. Available from: https://www.mdpi.com/1660-4601/18/17/9152 34501742 10.3390/ijerph18179152 PMC8431483

[47] ILO Estimates Over 1 Million Work-Related Fatalities Each Year [Internet]. 1999 [cited 2023 Aug 28]. http://www.ilo.org/global/about-the-ilo/newsroom/news/WCMS_007969/lang--en/index.htm

[48] Benti A, Kumie A, Wakuma S. Prevalence of occupational injury and associated factors among workers in large-scale metal manufacturing factories in Addis Ababa, Ethiopia. Ethiopian Journal of Health Development [Internet]. 2019 [cited 2023 Aug 28];33(2). Available from: https://www.ajol.info/index.php/ejhd/article/view/188850

[49] Mrema EJ, Ngowi AV, Mamuya SHD. Status of occupational health and safety and related challenges in expanding economy of tanzania. Annals of Global Health [Internet]. 2015 Dec 17 [cited 2023 Aug 28];81(4):538. Available from: https://annalsofglobalhealth.org/articles/10.1016/j.aogh.2015.08.021 26709286 10.1016/j.aogh.2015.08.021

[50] Premkumar A, Ying X, Mack Hardaker W, Massawe HH, Mshahaba DJ, Mandari F, et al. Access to orthopaedic surgical care in northern tanzania: a modelling study. World J Surg [Internet]. 2018 Oct [cited 2023 Aug 28];42(10):3081–8. Available from: http://link.springer.com/10.1007/s00268-018-4630-x 29696326 10.1007/s00268-018-4630-x

[51] BOA. Women in surgery–tanzania [Internet]. [cited 2023 Aug 28]. https://www.boa.ac.uk/about-us/diversity-and-inclusion/international-women-s-day/women-in-surgery-tanzania.html

[52] AAOS. ORTHOPAEDIC PRACTICE IN THE U.S. 2018 [Internet]. AAOS Department of Clinical Quality and Value; 2018. https://www.aaos.org/globalassets/quality-and-practice-resources/census/2018-census.pdf

[53] Tran Y, Jarrett J, Gardner S, Fernando J, Milliron M, Hong L. Long-term impact of interprofessional medical mission service trips in sierra leone. Front Med [Internet]. 2021 Sep 27 [cited 2023 Aug 28];8:742406. Available from: https://www.frontiersin.org/articles/10.3389/fmed.2021.742406/full 34646846 10.3389/fmed.2021.742406 PMC8502852

[54] Park J, Heo J, Kim WH. Establishing surgical care sustainability in sub-saharan africa for global child health: insights from pediatric cardiac surgical capacity-building programs in ethiopia and côte d’ivoire. Front Pediatr [Internet]. 2022 Jan 14 [cited 2023 Aug 28];9:806019. Available from: https://www.frontiersin.org/articles/10.3389/fped.2021.806019/full 35096714 10.3389/fped.2021.806019 PMC8795907

[55] Doctors Without Borders—USA [Internet]. [cited 2023 Aug 28]. Tanzania. https://www.doctorswithoutborders.org/what-we-do/where-we-work/tanzania

[56] Hendriks TCC, Botman M, Rahmee CNS, Ket JCF, Mullender MG, Gerretsen B, et al. Impact of short-term reconstructive surgical missions: a systematic review. BMJ Glob Health [Internet]. 2019 Apr [cited 2023 Aug 28];4(2):e001176. Available from: https://gh.bmj.com/lookup/doi/10.1136/bmjgh-2018-001176 31139438 10.1136/bmjgh-2018-001176 PMC6509599

[57] McClenaghan F, Fell M, Martin D, Smith G, McGurk M. Surgical mission planning in the developing world. International Journal of Oral and Maxillofacial Surgery [Internet]. 2013 Dec [cited 2023 Aug 29];42(12):1587–91. Available from: https://linkinghub.elsevier.com/retrieve/pii/S0901502713010618 24016548 10.1016/j.ijom.2013.07.748

[58] Khan T, Wahjoepramono E, Wahjoepramono P, Andrews R. Private healthcare initiatives in developing countries–Building sustainable neurosurgery in Indonesia and Pakistan. Brain and Spine [Internet]. 2023 [cited 2023 Aug 29];3:101729. Available from: https://linkinghub.elsevier.com/retrieve/pii/S2772529423000176 37383471 10.1016/j.bas.2023.101729 PMC10293311

[59] World Population Prospects The 2015 Revision. Key Findings and Advance Tables. Population Division: Department of Economic and Social Affairs; 2015.

[60] Kilama WL. The 10/90 gap in sub-Saharan Africa: Resolving inequities in health research. Acta Tropica [Internet]. 2009 Nov 1 [cited 2023 Aug 29];112:S8–15. Available from: https://www.sciencedirect.com/science/article/pii/S0001706X09002447 19695211 10.1016/j.actatropica.2009.08.015

[61] Nolte MT, Nasser JS, Chung KC. A systematic review of orthopedic global outreach efforts based on who-choice thresholds. Hand Clinics [Internet]. 2019 Nov [cited 2023 Aug 29];35(4):487–97. Available from: https://linkinghub.elsevier.com/retrieve/pii/S074907121930085X 31585610 10.1016/j.hcl.2019.07.015 PMC6779325

[62] Hariton E, Locascio JJ. Randomised controlled trials—the gold standard for effectiveness research: Study design: randomised controlled trials. BJOG: Int J Obstet Gy [Internet]. 2018 Dec [cited 2023 Aug 29];125(13):1716–1716. Available from: https://onlinelibrary.wiley.com/doi/10.1111/1471-0528.15199 29916205 10.1111/1471-0528.15199 PMC6235704

[63] Djurisic S, Rath A, Gaber S, Garattini S, Bertele V, Ngwabyt SN, et al. Barriers to the conduct of randomised clinical trials within all disease areas. Trials [Internet]. 2017 Dec [cited 2023 Aug 29];18(1):360. Available from: http://trialsjournal.biomedcentral.com/articles/10.1186/s13063-017-2099-9 28764809 10.1186/s13063-017-2099-9 PMC5539637

[64] Alemayehu C, Mitchell G, Nikles J. Barriers for conducting clinical trials in developing countries- a systematic review. International Journal for Equity in Health [Internet]. 2018 Mar 22 [cited 2023 Aug 29];17(1):37. Available from: doi: 10.1186/s12939-018-0748-6 29566721 PMC5863824

[65] Siegfried N, Volmink J, Dhansay A. Does South Africa need a national clinical trials support unit? S Afr Med J [Internet]. 2010 Jul 26 [cited 2023 Aug 29];100(8):521. Available from: http://www.samj.org.za/index.php/samj/article/view/3958 20822621 10.7196/samj.3958

[66] Exploring the current state of health research in sub-saharan africa [Internet]. [cited 2023 Aug 29]. https://www.rand.org/randeurope/research/projects/health-research-sub-saharan-africa.html

[67] Mtenga S, Masanja IM, Mamdani M. Strengthening national capacities for researching on Social Determinants of Health (Sdh) towards informing and addressing health inequities in Tanzania. International Journal for Equity in Health [Internet]. 2016 Feb 9 [cited 2023 Aug 29];15(1):23. Available from: doi: 10.1186/s12939-016-0308-x 26860192 PMC4746920

