# Supplementary material for: Orthopaedic and trauma research in Tanzania: A scoping review
Source: PLoS One. 2024 Jun 5;19(6):e0304218. doi: 10.1371/journal.pone.0304218 (PMC11152290; doi:10.1371/journal.pone.0304218)
Supplement: S1 File — (PDF) [file pone.0304218.s002.pdf]

Search Strategy

<u>PubMed</u>:
("Orthopaedics"[MeSH Terms] OR "Orthopaedics"[All Fields] OR "Musculoskeletal Diseases"[MeSH Terms] OR "Musculoskeletal Diseases"[All Fields] OR "Wounds and Injuries"[MeSH Terms] OR "Wounds and Injuries"[All Fields]) AND ("Tanzania"[MeSH Terms] OR "Tanzania"[All Fields])

<u>Medline</u>:
exp Orthopaedics/ OR Orthopaedics.tw.
exp Orthopaedics/ OR Orthopaedics.tw.
exp Musculoskeletal Diseases/ exp
Wounds and Injuries/ exp Fractures,
Bone/ exp Arthritis/ exp Joint Diseases/
exp Spinal Diseases/ exp Trauma,
Nervous System/
1 OR 2 OR 3 OR 4 OR 5 OR 6 OR 7 OR 8 OR 9 exp
Tanzania/
10 AND 11

<u>CINAHL</u>:
(("Orthopaedics" OR "Orthopaedics" OR "Musculoskeletal Diseases" OR "Musculoskeletal Injuries" OR "Trauma" OR "Fractures" OR "Wounds and Injuries" OR "Arthritis" OR "Osteoporosis") AND "Tanzania")
